# Practitioners’ perspective: a mixed-methods study on dealing with suicidality from the perspective of oncological healthcare professionals

**DOI:** 10.1007/s00432-025-06106-z

**Published:** 2025-01-28

**Authors:** Tamara Schwinn, Judith Hirschmiller, Jörg Wiltink, Rüdiger Zwerenz, Elmar Brähler, Manfred E. Beutel, Mareike Ernst

**Affiliations:** 1 Department of Psychosomatic Medicine and Psychotherapy, University Medical Center of the Johannes Gutenberg-University Mainz, Untere Zahlbacher Str. 8, 55131 Mainz, Germany; 2 University Cancer Center Mainz (UCT), University Medical Center of the Johannes Gutenberg-University Mainz, Mainz, Germany; 3 Department of Medical Psychology and Medical Sociology, University Medical Center of Leipzig, Leipzig, Germany; 4 Department of Clinical Psychology, Psychotherapy and Psychoanalysis, Institute of Psychology, University of Klagenfurt, Klagenfurt am Wörthersee, Austria

**Keywords:** Suicide prevention, Healthcare professionals, Cancer patients, Oncology, Psycho-oncology, Mixed-methods

## Abstract

**Purpose:**

Healthcare professionals (HCPs) play a critical role in suicide prevention and clinical guidelines recommend inquiring about suicidality as part of medical history and diagnosis. Emerging evidence indicates a lack of implementation of such policies in clinical practice. However, to date, no comprehensive mixed-methods study has examined this issue in the field of oncology.

**Methods:**

A preregistered mixed-methods study was conducted with oncological HCPs (*N* = 20) from various professions, using semi-structured interviews and validated questionnaires. Employing an explorative theory-generating approach, qualitative content analysis was applied to the interviews. The different data sources are integrated and contrasted. Comparisons according to sociodemographic variables (profession, age, and gender) and frequency distributions were used to examine the questionnaire data.

**Results:**

Most HCPs reported direct or indirect experiences with suicidality in cancer patients. Nineteen HCPs did not routinely explore suicidality, of whom five reported not inquiring about it at all. Those who explored suicidality were more confident, less emotionally overwhelmed and reported higher subjective knowledge. HCPs also differed regarding their endorsement of suicide myths.

**Conclusion:**

The study highlights difficulties with active suicide exploration and differences among HCPs. Integrating these findings into education and training could improve HCPs’ skills and reduce disparities, supporting successful suicide prevention.

**Supplementary Information:**

The online version contains supplementary material available at 10.1007/s00432-025-06106-z.

## Highlights

Mixed-Methods study on suicide prevention from oncology experts’ perspective.

Experts experience different manifestations of suicidality in cancer patients.

Recommendations to actively explore suicidality are not adequately followed.

Differences in knowledge, confidence, and stigmatization.

Common, but also diverging agreement with suicide myths.

**Supplementary Information:**

The online version contains supplementary material available at 10.1007/s00432-025-06106-z.

## Introduction

Suicide remains a leading cause of death globally, claiming over 720,000 lives annually (World Health Organization 2024), with 10,300 suicides recorded in Germany in 2023 (Destatis 2024). Research indicates that physical illness such as cancer significantly heightens the risk of suicidal thoughts and behaviors (STBs). Reviews identify an up to 3.5-fold increased risk for suicidality among cancer patients compared to the general population (Amiri and Behnezhad 2019; Robson et al. 2010; Zaorsky et al. 2019). Given this elevated risk, suicide prevention for cancer patients is of paramount importance.

Current guidelines, including the National Cancer Plan, emphasize healthcare professionals’ (HCPs) important role as gatekeepers. The recommendation requires HCPs of all disciplines involved in cancer care to *actively explore* STBs (Deutsche Krebsgesellschaft; Deutsche Krebshilfe; AWMF 2023; Nationaler Krebsplan 2017). This is crucial as many patients do not readily disclose STBs due to shame, internalized stigma (Hom et al. 2017) or fear of involuntary hospitalization. Especially men find it hard to disclose their distress due to the influence of conformity with traditionally masculine norms (Jerant et al. 2019), which is dangerous as male cancer patients are particularly vulnerable to suicidality (Schwinn et al. 2024) and men are also overrepresented among those dying by suicide (Turecki et al. 2019).

Actively engaging patients in conversations about suicidality has proven beneficial (Calear and Batterham 2019), as a professional who takes their suffering seriously and reacts empathically can provide relief. This is the basis of a good therapeutic alliance (also working/helping alliance; see Bordin (1979), the best predictor of psychotherapy outcome (Fluckiger et al. 2018). A strong patient-oncologist alliance protected against suicidal ideation (Trevino et al. 2014). Expert advice highlights using appropriate language in such conversations (Saini et al. 2024). Also, the use of closed or negative questions could discourage disclosure, limiting the potential for more in-depth conversations (McCabe et al. 2017).

Despite these insights and an increased understanding of suicide prevention as a professional imperative (Jobes and Barnett 2024), research indicates that suicidality is not adequately addressed in clinical practice: A vignette study found that the majority of HCPs do not routinely screen for STBs (Hooper et al. 2012). Almost half of those who died by suicide had seen a primary care provider within a month of their death, underscoring the complexity of addressing STBs, including challenges in openly discussing them, recognizing the risk, and intervening (Luoma et al. 2002). Similarly, 25% of older cancer patients had HCP contact in the week preceding their death by suicide (Miller et al. 2008).

Evidence highlights that addressing STBs can be challenging due to the multifaceted emotional experiences of HCPs when managing suicidal patients. These experiences are described as adverse, fortunate, or bittersweet (Ferracioli et al. 2023) and can lead to emotional responses such as distress, guilt, surprise, and feeling emotionally overwhelmed (Granek et al. 2019a). Even with risk assessment tools, many HCPs report insecurity in these situations (Turner et al. 2023). In a German study, 72% of HCPs felt insecure, 20% overwhelmed, and 50% lacked adequate knowledge in dealing with suicidality, with variation across professional groups (psychotherapists reporting the lowest rates)(Senf et al. 2020). Similarly, a mixed-methods review highlighted substantial differences in HCPs’ suicide-related knowledge (Dillon et al. 2020). Similar patterns were observed in various studies, where a lack of knowledge (Wardig et al. 2022) and a scarcity of specific skills contributed to difficulties and uncertainty in managing suicide risk (D’Alton et al. 2021; Ozturk and Hicdurmaz 2023).

Besides professionals’ anxieties, the stigma surrounding STBs further complicates care, particularly for cancer patients with mental health issues (D’Alton et al. 2021). Patients with previous suicidal behaviors also face stigma from non-mental health providers, hindering appropriate referrals and treatment (Frey et al. 2016). This is concerning as most who die by suicide lack contact with mental health services in the year preceding their death (Appleby et al. 2018). Stigmatization is also associated with endorsing suicide myths, i.e., disproven, unhelpful beliefs about suicidality/suicidal individuals (such as that discussing suicidality may trigger it, or that those expressing suicidal thoughts are seeking attention but not actually at risk). These misconceptions foster inadequate responses and undermine evidence-based recommendations that any suicidal crisis requires conversation, relationship-building, and prompt intervention (O’Conner 2016).

Despite the evidence of suicide risk in cancer patients and the critical role of oncological HCPs in suicide prevention, so far, no study has thoroughly examined their knowledge, attitudes, and suicide prevention behavior. To our knowledge, this study represents the first mixed-methods investigation of German HCPs’ experiences, linking their subjective knowledge and endorsement of suicide myths with their behavior toward oncological patients.

### Aims and objectives

In particular, we aim to answer the following research questions:

1. What experiences do oncological HCPs have with suicidality in cancer patients?
2. Do oncological HCPs actively inquire about suicidality? If yes, how?
3. How do oncological HCPs manage suicidality?
4. Are there differences in relation to HCPs’ professional group, age, and gender?

## Materials and methods

### Study design and participants

The study employed a mixed-methods design combining qualitative, semi-structured interviews with quantitative data collection using a standardized questionnaire. This approach allowed to examine HCPs’ specific practices, coping strategies, and workplace challenges while identifying potential discrepancies between self-reported and surveyed behaviors. The study’s aims and procedure were preregistered using the Open Science Framework where the materials (including the interview guide and questionnaire) are available (https://osf.io/4c6jr).

Participants (*N* = 20) included HCPs from the oncology unit of a large University Medical Center (certified as a Comprehensive Cancer Center) and associated departments (e.g., the psycho-oncology section of the Department of Psychosomatic Medicine and Psychotherapy). Participants were recruited via internal mailing lists, flyers, and relaying through department directors/other staff. Inclusion criteria were active engagement in cancer patients’ care and sufficient knowledge of German. We aimed to include various professional backgrounds. Participants were not paid. They were informed about the study’s aims, handling of personal data and anonymity of responses and all provided written informed consent prior to the study. The study contents and procedures were approved by the ethics committee of the Rhineland-Palatinate Chamber of Physicians (No. 2023–16975).

### Interview procedure

The interviews, averaging *M*(*SD*) = 39.47(8.35) minutes (range: 21.83–52.02 min) were conducted by one of two project members (both psychologists and psychotherapists in advanced training, one certified as psycho-oncologist). Interviews were audio-recorded and subsequently transcribed according to established rules (Kuckartz 2010). HCPs were not provided with response categories, encouraging them to reflect on their everyday working lives. The sample size was determined following the theoretical sampling of qualitative studies to achieve representativeness and consistency (Corbin and Strauss 1990). Recruitment and interviews were stopped after reaching a saturation point, in line with common procedures in qualitative research. According to Guest et al. (2006), this is the point at which no new information/new categories concerning the research question can be observed in the data. Our sample size is in line with common observations (Guest et al. 2006).

The semi-structured interview guide was designed based on the empirical literature, piloted with a certified psycho-oncologist and psychologist and refined with the help of experienced scientist-practitioners. Congruent with an exploratory, theory-generating approach, redundant questions were shortened or summarized during data collection. Topics included: participants’ oncology work experience, encounters with suicidality, suicide risk assessment, and management practices. Additional findings regarding barriers, optimization strategies, and training preferences are reported separately (Schwinn et al., under review).

### Questionnaire

After the interview, the questionnaire was handed out. This order was chosen to prevent the questionnaire’s content from influencing the answers to the open questions in the interview. HCPs could either complete it immediately or take it with them (in case of time pressure due to their work schedule), send it, and hand it in at another time. The questionnaire took about 15 min and assessed sociodemographic (e.g., age, gender, professional group/experience) information as well as the following variables:

*Subjective knowledge.* Three items (Senf et al. 2022) assessing self-perceived knowledge regarding suicidality in general, in cancer patients, and regarding legal regulation on suicide prevention, from 1=“insufficient” to 4=“very good”.

*Confidence.* The Confidence in talking about suicidality with patients was rated with one item “How confident do you feel talking to patients about suicidal thoughts?” (used in a previous German study (Senf et al. 2022) with a scale from 1=“very unconfident” to 4=“very confident”.

*Being emotionally overwhelmed*. We used a previously employed item by Senf et al. (2022) (from 1=“very overwhelmed” to 4=“not at all overwhelmed”): “If a patient substantiates suicidal desire in conversation, do you feel overwhelmed?”.

*Self-efficacy.* Self-efficacy was assessed with the ASKU (German for General self-efficacy short scale (Beierlein et al. 2013)) which comprises three items (e.g., “I can rely on my own abilities in difficult situations”). Answers were given on a scale from 0=“does not apply at all” to 5=“applies completely”. It has previously shown good internal consistency (ω = 0.81–0.86) (Beierlein et al. 2013).

*Suicide myths.* Suicide myths were assessed with nine items from Senf et al. (2022) and three items created on the basis of the described suicide myths by O’Connor (2021), e.g. “Asking patients about suicidality can cause/may trigger or reinforce their suicidal wishes” with a scale from 1=“I don´t agree at all” to 4=“I agree completely”.

*Stigmatization*. Stigmatization was assessed with the Stigma of Suicide Scale (SOSS-SF-D, German version (Ludwig et al. 2020)). Using sixteen items, people affected by suicide are rated on three subscales (depression/isolation: e.g., “lonely”; glorification/normalization: e.g., “strong”; and stigmatization: e.g., “pathetic”). Participants respond on a scale from 1=“strongly disagree” to 5=“strongly agree”. Internal consistency was acceptable (α = 0.71).

### Analysis

A qualitative content analysis of the interview transcripts using the software MAXQDA 2022 R22.7 V5 was performed (Kuckartz and Rädiker 2022), resulting in a deductively (using literature and interview guide) and inductively (using the transcripts for new categories) developed category system, which was applied using line-by-line coding. At least 30% of the transcripts were checked by the intercoder (another research team member) for conformity. Disagreements were discussed until a consensus was reached or decided by a senior researcher. This approach was also applied to the analysis process.

The questionnaire data were analysed with R statistics version 4.3.1 regarding sociodemographics with the constructs of interest by frequency distributions, group comparisons (one-way-ANOVAs), and visualizations. A Mann-Whitney-U-Test was performed for differences between two groups and a Kruskal-Wallis test for more than two groups as well as pairwise comparisons using Dunn’s test with Bonferroni correction.

To integrate and compare the qualitative and quantitative results, a side-by-side display was constructed, examining quotes from individuals with low vs. high scores on the variables of interest.

## Results

### Study sample

The study included 20 HCPs with different professional backgrounds (physicians, psychologists, [specialist] nursing staff, and pastoral care; see Table 1). Participants’ ages ranged from 28 to 60 years (*M* = 41.45; *SD* = 9.7) and their professional experience from 5 months to 20 years. The category system for the initial three questions is available in Supplementary Tables 1 and is described below.

**Table 1:** Sample characteristics and reported professional experiences

Sociodemographic characteristics	All HCPs (*N* = 20)
Gender *N* (%)	
Men	6 (30)
Women	14 (70)
Age Mean (SD)	41.45 (9.70)
Age Range [in years]	28–60
Highest degree/level of education *N* (%)	
Certificate of Secondary Education	2 (10)
High school (degree/graduation), polytechnic degree	1 (5)
Vocational/professional training, apprenticeship	5 (25)
Master’s Degree, state examination	8 (40)
Doctorate (e.g., PhD, EdD), licensed physician/therapist	4 (20)
Professional group *N* (%)	
Physician	5 (25)
Psychologist (psycho-oncologist/in training to psychotherapist/palliative psychologist)	5 (25)
Nursing staff	4 (20)
Specialist nursing staff (Study Nurse/oncological, medical assistant)	4 (20)
Pastoral care	2 (10)
Time in oncological practice *N* (%)	
5 months – 2 years	1 (5)
3 years – 9 years	5 (25)
10 years – 14 years	4 (20)
15 years – 19 years	1 (5)
≥ 20 years	5 (25)
Not applicable because HCPs worked across sectors	4 (20)
Frequency of contacts with suicidal patients *N* (%)	
None	6 (30)
1 – 3 times/year	10 (50)
≥ 3 times/year	4 (20)
Number of suicide deaths (of own patients) *N* (%)	
1 – 2	6 (30)
5	1 (5)
10	1 (5)

Note. The professional group shows their highest or for the study relevant position

### Question 1: Experiences with suicidality in cancer patients

In the questionnaire, six HCPs indicated having no contact with suicidal cancer patients, while ten HCPs attended to one to three, and four HCPs attended to more than three suicidal patients annually (Table 1). Moreover, eleven HCPs had private experiences with suicidality in family or friends (*N* = 4) or relatives (*N* = 1). Three each reported their own experiences with suicidality (*N* = 3) or did not provide more information (*N* = 3), while nine reported no private experiences with suicidality.

Despite initial claims of limited experience (e.g., due to lacking experience in the field), all HCPs reported direct or indirect experiences with different manifestations of suicidality (e.g., in own patients or colleagues’ patients, reading about it in the documentation, or hearing about a patient’s suicide from the patient’s relatives). These described experiences can be differentiated into four categories: (1) passive death wishes/suicidal thoughts, (2) death and dying, (3) stopping life-sustaining actions/suicidal actions and suicide deaths and (4) positive experiences.

All professional groups reported encounters with (1); e.g., “One in ten patients, maybe even a little more (…) expresses at least passive death wishes.” (EI12_psychologist). While some of the HCPs cited somatic symptoms (e.g., pain) as contributing factors for developing suicidal thoughts, paradoxically, improvements in physical symptoms were mentioned triggering suicidal thoughts as well. The common mention of (2) highlights the difficulty in distinguishing between suicidal crises and conversations around assisted suicide/end-of-life care (e.g., “(…) people who are in a hospice [are] very different [from non-palliative patients and how] (…) they deal with impending death.” (EI20_pastoral care)). One psychologist said: “[The patients] want to live and are afraid of dying” (EI17). However, they also reported that one of their patients first expressed thoughts of weariness of life and two weeks later acute suicide risk had to be reassessed. Psychologists, (specialist) nursing staff and pastoral carers shared experiences with (3) of their own patients (e.g., “From time to time it happens; this year it has already happened once (…). She couldn’t cope psychologically and took her own life.” (EI3_specialist nursing staff)). Stopping life-sustaining actions included food refusal or avoidance of treatment. HCPs in the intensive care unit were most likely to encounter patients who attempted suicide.

Some patients had been referred to psychiatric wards, and their suicide deaths occurred post-discharge from these. But this was not always the case. The impact of these deaths was significant, affecting both patients and staff (e.g., “It made waves when a patient witnessed someone jumping off the roof (…) that’s something you don’t need in a situation that’s already burdensome” (EI13_psychologist)). In addition, twelve of the interviewees reported witnessing or hearing about suicide deaths (in their own ward, other wards, or patients unknown to them). Some of the reports concerned certain patients who died by suicide in the oncological ward. Despite the noted challenges, some psychologists reported (4) discussing suicidality with their patients, describing patients’ relief and improved mood after conversations.

### Question 2: Asking about suicidality

Twelve HCPs reported discussing suicidality with patients, and 14 reported asking about it under certain conditions (e.g., patient expression, screening results, or intuition) (Supplementary Table 2). However, most (*N* = 19) did not *routinely* explore suicidality, of whom five did *not* explore suicidality *at all*. Although the HCPs had a concept of potential protective/risk factors or particularly vulnerable groups (e.g., according to the level of social support, financial resources or gender (with men less likely to disclose suicidality) and age), naming specific criteria was difficult for the HCPs, as well as reporting the questions they used. Standard questions included *explicit* (“Do you have thoughts/plans of taking your own life?” (EI13_psychologist) or “Are you acutely suicidal? Is there an acute danger?” (EI4_physician)) to *non-explicit questions* (*“*What kind of images come to mind when you think of peace?” (EI19_pastoral care)). Suicide exploration was understood as a conversation, using “several phases of exploration” to “get an understand what is behind it [meaning patients’ initial reports]” (EI12_psychologist), reflecting concern for patient dignity and context.

### Question 3: Managing suicidality

Nineteen HCPs had reliable referrals (colleagues, supervisors, psycho-oncology, psychiatry, pastoral care) for patients expressing suicidality (e.g., “I consult with my psycho-oncological team - that would be my first point of contact, alongside the physicians” (EI13_psychologist), “We have the psycho-oncology [as support for the team]. But pastoral care is also always requested, as we have a 24-hour emergency call service.” (EI19_pastoral care) and “[My contact persons are] my colleagues. We have exchange” (EI16_specialist nursing staff)). A mere 10 HCPS mentioned own strategies, e.g. specific interventions such as a no-suicide contract (*N* = 4) and withdrawal of lethal medication (*N* = 2), or more general approaches such as perspective-talking (*N* = 1), involving persons of trust or the patients’ social network (*N* = 1), administration of medication (*N* = 3), regular checking and conversation (*N* = 6), normalization/psychoeducation (informing patients that experiencing thoughts of weariness of life is not uncommon after receiving a life-altering diagnosis) (*N* = 2), and resource activation with imagination (*N* = 1). Reactions to *suicide deaths* varied widely from team discussions to avoidance of the topic, with a focus on future prevention or guilt over perceived mistakes, including difficult feelings like shock, sadness, surprise, and emotional blunting (“I was a bit shocked, and then thought, should I have seen that somehow? [after learning about a patients’ suicide]” (EI7_nursing staff)).

### Question 4: Differences in relation to sociodemographics

**Fig. 1:**
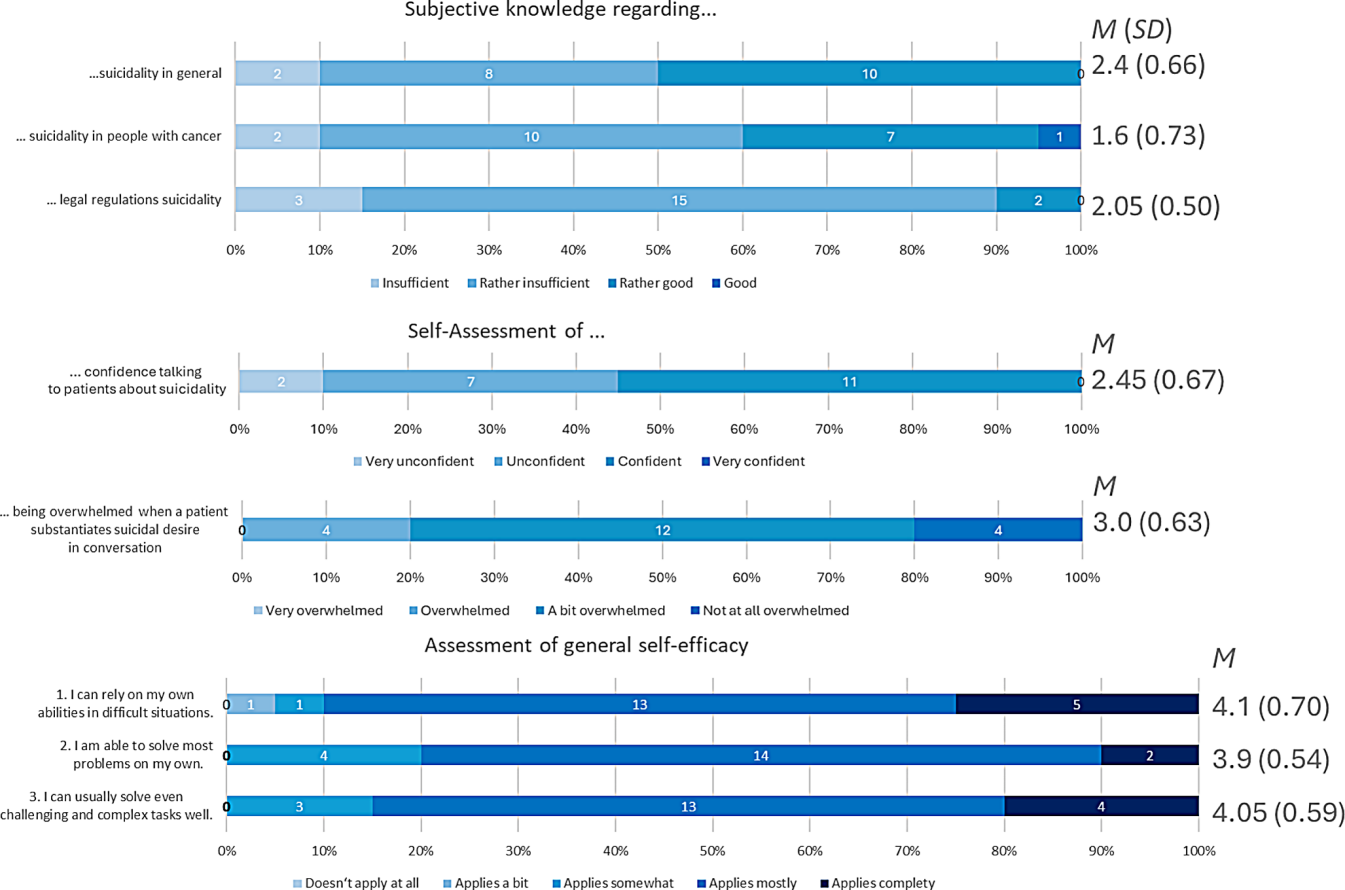
Piled bar charts of subjective knowledge, confidence, being emotionally overwhelmed and self-efficacy. This figure shows the frequency of each answer of the 20 HCPs as well as mean and standard deviations of responses to each question. The different professional groups rated their subjective knowledge as lower for the more specific kinds of knowledge. Most HCPs rated themselves as “a bit overwhelmed” in encountering suicidal desire. The HCPs rated their self-efficacy as high

**Fig. 2:**
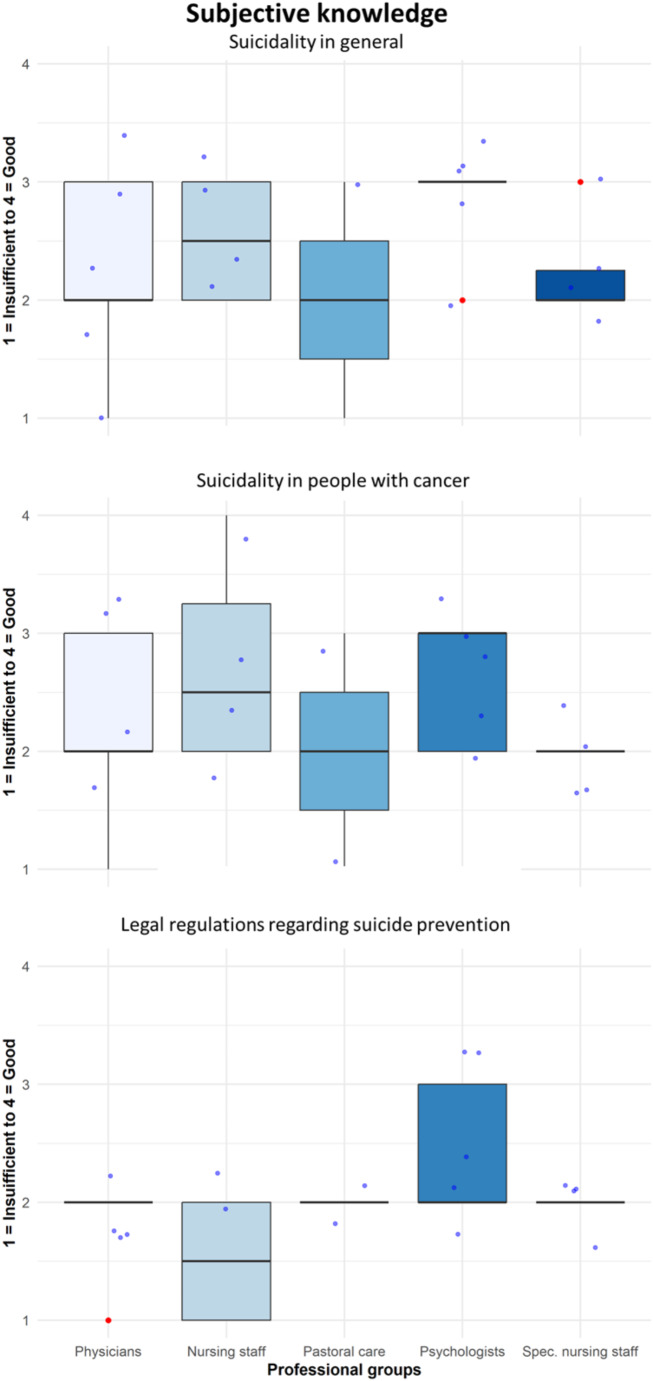
Boxplots of subjective knowledge by professional groups. The boxplots show differences in three levels of subjective knowledge regarding the professional groups. The more specific the questioned topic, the lower the mean value of subjective knowledge

*Subjective knowledge.* The interviews revealed the lack of a consistent approach to assessing suicidality. Figure 1 indicates a decline in subjective knowledge the more specifically HCPs were asked about suicidality. For instance, Fig. 2 shows differences across professional groups: psychologists rated their general knowledge the highest, yet it declined with increasing focus on suicidality, while nursing staff perceived themselves the lowest in legal knowledge. A Kruskal-Wallis test revealed no significant differences between professional groups or genders. Age was not associated with knowledge.

Here, the integration of interview and questionnaire data yielded further insights, with one HCP rating their knowledge as “good” within the questionnaire, but casting doubt on the feasibility of assessing suicide risk.

*Confidence.* Most participants felt “unsure” and “certain” (Fig. 1). The Kruskal-Wallis test showed a difference between professional groups (χ^2^(4) = 10.09, *p* =.039), but post-hoc tests found no significant differences. No significant difference was shown in age or gender.

**Fig. 3:**
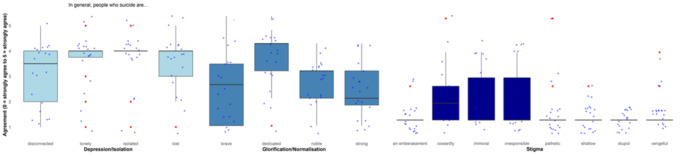
Boxplots of the estimation of people who die by suicide (the German version of the Suicide of Suicide Scale (SOSS-SF-D)). This boxplot shows the results of the German version of the Suicide of Suicide Scale (SOSS-SF-D). It shows higher agreement with the Depression/Isolation scale (M(SD) = 3.43(1.17)) and the Glorification/Normalisation (M(SD) = 3.45(1.13)) scale. There was less agreement with the items of the Stigma Scale (M(SD) = 1.34(0.68)

*Being emotionally overwhelmed.* Most HCPs felt “a little overwhelmed” (Fig. 3). There were no significant differences between professional groups, age or gender.

*Self-efficacy.* Self-efficacy was rated at *M*(*SD*) = 4.02(0.51) (Fig. 1), with physicians scoring the highest. No differences were found between professional groups, age or gender.

*Suicide myths.* Suicide myths were frequently mentioned in the interviews, either to contradict or reinforce them. High agreement with suicide myths was observed in the questionnaire, especially for the statements “Most patients who die by suicide suffer from depression" (myth 1) and “(…) some kind of psychiatric disease” (myth 2). Many HCPs identified mental disorders such as depression as a key risk factor for suicidality in the interviews. Myth 9 was supported by four HCPs in the interviews. In the case of myth 7, statements from interviews showed that HCPs supported it. At the same time, there were also divergences between the statements made in the interviews and the level of agreement with these myths in the questionnaire (Table 2), for instance, in the sense that one HCP disagreed with the myth (that patients who announce their suicide do not follow through) when it was presented within the questionnaire, but still narrated it in the interview (stating “barking dogs don’t bite”). Endorsing myth 7 correlated negatively with age (*r* = -.55, *p* = .013). The agreement to other myths did not differ between age or gender.

**Table 2:** Suicide myths – comparison of questionnaire responses and interview

Person	Questionnaire response: Agreement with the myth?	Interview quotes / summarized statements	Divergence/Convergence between information from interview vs. questionnaire
	Myth 7: Most patients who announce their suicide do not follow through		
3	No agreement	Those who say it don’t do it (“barking dogs don’t bite”).	Divergence
4	Agreement	Some patients [report suicidality] for attention.	Convergence
	Myth 8: Asking patients about suicidality can cause / may trigger or reinforce their suicidal wishes		
1	Agreement	I am concerned that suicidal thoughts may be triggered by inquiring about suicidality.	Convergence
2	No agreement	It is nonsense to think that you would trigger suicidality by asking about it.	Convergence
3	Agreement	I would be afraid to basically give someone guidance to harm themselves by asking about suicidality	Convergence
6	Agreement	There is a fear of giving someone the idea of harming themselves by asking about suicidality.	Convergence
8	No agreement	I am worried that asking about suicidality will give a patient this idea.	Divergence
9	No agreement	No, I do not think that you can trigger suicidality by asking.	Convergence
10	Agreement	I [do not ask about suicidality because I] do not want to trigger anything negative in the patient; and I would blame myself if this was the case.	Convergence
11	No agreement	I do not think that asking about suicidality gives anyone the idea to engage in suicidal behavior.	Convergence
14	No agreement	Asking about suicidality has something protective about it.	Divergence
16	Agreement	I am worried that you plant the idea by asking and, e.g., persons with schizophrenia or anxiety will “lose it” and become suicidal if you bring up suicidality.	Convergence
17	No agreement	I think that others, e.g., doctors, are possibly afraid to “open a can of worms” by asking about suicidality.	Convergence
18	No agreement	I previously thought that I might give someone the idea by asking about suicidality, but I do not think so anymore.	Convergence
20	Agreement	I am worried that asking about suicidlity will trigger it.	Convergence
	Myth 9: We cannot stop anyone from dying by suicide		
2	Agreement	I do not think that you cannot stop a person, because they will do it anyway.	Convergence
9	No agreement	In certain individuals, suicide cannot be prevented.	Divergence
14	No agreement	Someone who has a firm decision will also succeed [in suicide]. There is a percentage of people who cannot be saved.	Divergence
19	No agreement	If someone wants to kill themselves, they will do it (in the sense that they say: “that’s my conviction”)	Divergence

Note. The table juxtaposes the questionnaire and interview data. Originally, the response format within the questionnaire ranged from 1 (“Do not agree at all”) to 4 (“Completely agree”). To ease interpretation and comparisons, it was transformed to a binary format (1–2: No agreement; 3–4: Agreement)

For myth 8, interview responses were mixed, with discrepancies between interviews and questionnaires within HCPs (Table 2). Some HCPs raised knowing about the concern that asking about suicidality might trigger it, but also stated that they disagreed, while others agreed with it. The Kruskal-Wallis test revealed significant differences between professional groups (χ²(4) = 13.66, *p* =.008), but post-hoc analysis showed no significant differences.

*Stigmatization.* Stigmatization of suicidality was evident in interviews, with many HCPs avoiding the topic due to preconceived notions, such as patients being too old to be asked about suicidality, the topic not relevant, or suicide exploration is not preventive. The questionnaire showed comparatively higher stigmatization scores in depression/isolation and glorification/normalization subscales (Fig. 3). The Kruskal-Wallis test showed significant differences between professional groups (χ^2^(4) = 10.12, *p* =.039), though no post-hoc differences emerged.

*Relation*s. There was no significant difference between HCPs who explore and those who do not explore suicidality regarding self-efficacy or stigmatization, but there was a difference regarding the statement that in oncological patients, suicidal thoughts were a means for regaining control (interview: W = 15.5, *p* =.046 (*d* = -0.98); questionnaire: W = 14.5, *p* =.010 (*d* = 1.44)) and the statement that it was a sign for suicidality if an oncological patient aborts/ceases therapy (questionnaire: W = 17.5, *p* =.010 (*d* = -1.48)).

There were differences in confidence (interview: W = 10.5, *p* =.009 (*d* = -1.48); questionnaire: W = 18.5, *p* =.018 (*d* = -1.13)), being emotionally overwhelmed (interview: W = 13.5, *p* =.018 (*d* = -1.43), and subjective knowledge about suicidality (interview: W = 12.5, *p* =.018 (*d* = -1.34)) between those exploring and not exploring suicidality; with the former reporting greater confidence, being less emotionally overwhelmed, and knowing more.

In general, confidence (*r* =.59, *p* =.006), self-efficacy (*r* =.53, *p* =.017), and stigmatization (*r* =.53, *p* =.017) were associated with being (less) emotionally overwhelmed.

## Discussion

This investigation aimed to fill the research gap of examinations of how German oncological HCPs manage suicidality, comparing qualitative and quantitative data on key aspects such as knowledge, confidence, being emotionally overwhelmed, self-efficacy, suicide myths, and stigmatization. By integrating qualitative and quantitative data, this research gives insight into the interplay of these factors and HCPs’ subjective experience.

The findings can be related to the three dimensions of the therapeutic alliance as postulated by Bordin (1979), even if suicide prevention efforts transcend the specific psychotherapeutic setting.

The first dimension, *“Agreement on goals*,*”* highlights the importance of mutual agreement between patients and HCPs. In this context, the primary goal should be to provide effective support to the patient. However, our findings indicate a lack of goal alignment if HCPs do not actively explore STBs as this counteracts patients’ wishes to experience relief. Professionals from all groups had shared various experiences with (private and) patient STBs including suicide deaths among their patients. Although encompassing the full range of suicidality, in line with existing literature (Lauterbach and Kienast 2009), a systematic inquiry into suicidality was not part of their routine. Instead, HCPs often relied on patients seeking additional care or on high distress screening scores (not specific for suicidality).

The second dimension, *“Tasks”* refers to specific actions undertaken by HCPs and patients, with clearly defined roles and responsibilities. While this dimension was only partially fulfilled, one critical task for effectively managing suicidality is for all HCPs working with cancer patients to systematically assess suicide risk. This requires the use of clear, standardized criteria and well-defined strategies. In addition to unspecific risk factors (e.g., male gender), some participants intuitively mentioned protective factors such as social support (Du et al. 2020). Although relying on intuition can lead to clinical bias and misdiagnosis (Granek et al. 2018), also keeping protective factors in mind is noteworthy (and contrasts the literature’s heavy focus on risk factors (Ernst et al. 2024)). Participants mentioned prevention strategies included removing lethal means (e.g., medication) (Hawton et al. 2024) and no-suicide contracts (although they are less effective than crisis-response plans (Lengvenyte et al. 2021) and safety planning (Nuij et al. 2021)).

The third dimension “*Bond*” emphasizes the importance of a trusting and supportive interpersonal relationship. An absence of emotional connection can hinder effective communication, including a thorough and sincere inquiry about STBs. In contrast to previous research (McCabe et al. 2017; Saini et al. 2024), HCPs reported using open-ended and in parts vague questions. This seems problematic as they could be hard to answer, especially for cognitively impaired patients (Pendergrass et al. 2017). Adding to this, gradually addressing STBs in a longer conversation/over multiple appointments (though well-intentioned to foster trust), may be challenging – given the time constraints in clinical practice – and miss out on acute crises. Also, not asking about STBs because of fear that it might hurt the emotional bond might achieve the opposite as it prevents patients from making the positive experience (as reported by the psychologists) that open conversations about STBs offer relief (Calear and Batterham 2019). Furthermore, stigmatization, minimization and endorsement of suicide myths all present obstacles to empathic, emotional connection.

Thus, the implementation gap of suicide prevention recommendations in clinical practice is at odds with all three dimensions of a good therapeutic alliance. Specific barriers highlighted by our findings are a lack of knowledge, low confidence, feelings of being overwhelmed, as well as stigmatization and misconceptions. Not surprisingly, participants who explored suicidality felt more confident, less emotionally overwhelmed and rated their subjective knowledge higher. This fits well with the evidence showing that HCPs who perceive themselves as competent in assessing suicidality are less willing to work with suicidal individuals (Groth and Boccio 2019).

A major challenge regarding lack of knowledge was difficulty in distinguishing STBs from other death-related issues (end-of life/palliative care), a complexity also reported by Granek et al. (2019b). This difficulty may be further compounded by contextual factors specific to Germany, such as the availability of assisted suicide in neighboring countries like Switzerland and ongoing debates on changing regulations around assisted suicide. Moreover, depression and cancer-related symptoms often overlap, further complicating the assessment of STBs, as noted by both HCPs and supported by empirical studies (Schellekens et al. 2020).

However, divergences between interview and questionnaire responses may reflect social desirability bias or internal conflicts within HCPs, many of whom understood that discussing suicidality can save lives but still agreed with myth 8 that asking patients about suicidality could trigger it. Thus, there could potentially be a gap between their fact-based knowledge (that exploring suicidality is not dangerous) and their behavior (not exploring suicidality due to diffuse worries/anxiety).

Regarding stigmatization, a heterogenous picture emerged. Our results showed similar scores as in the population sample surveyed to validate the scale (Ludwig et al. 2020). Although suicidal patients were not generally stigmatized, they were often perceived in a glorified or depressed light which mirrors the high agreement with suicide myths 1 and 2 (suffering from depression/psychiatric disease). The potentially detrimental impact of the high agreement in myth 7 (people announcing suicidality do not follow through) is also shown in a study by Wee et al. (2020) in which HCPs did not believe their patients when they reported suicidality.

*To support* HCPs in actively exploring suicidality and supporting their patients in line with *the three dimensions constituting a good alliance*, it is crucial to provide them with comprehensive resources, including appropriate training, clear guidelines, validated tools, supervision, and referral options. These can help create an environment where suicidality can be effectively identified and addressed, while also acknowledging and managing the emotional challenges inherent in suicide prevention.

Suicide prevention training can improve self-perceived knowledge about suicide and related skills (Siau et al. 2018; Solin et al. 2021; Stuber et al. 2023), enhance self-efficacy (Osteen et al. 2017; Siau et al. 2018), increase confidence in managing suicidal patients (Muehlenkamp et al. 2023; Stuber et al. 2023) and reduce stigmatization and suicide attempts in affected individuals (Demesmaeker et al. 2023). These improved factors translate into greater willingness to help suicidal patients and positively influence practice behavior (Osteen et al. 2017; Siau et al. 2018). These training courses should include active elements (such as role plays) and clearly prepared knowledge transfer introducing guidelines for risk assessment and suitable standardized screening tools – such as the Columbia-Suicide Severity Scale by Posner et al. (2011), which aids risk detection across settings (Riblet et al. 2022). Our findings underscore the importance of a reflection and engagement with one’s own emotional reactions, stereotypical thinking, and “blind spots” (such as misconceptions, anxiety and avoidance) in HCPs’ training and also supervision.

Furthermore, the emotional burden of managing suicidality could be intensified by the absence of supervision and team discussions (or their focus on mistakes), which support the perception that suicide prevention is a shared responsibility and professional task (Jobes and Barnett 2024). Creating space for open discussions within teams (including furthering conversations around dying with dignity and shared decision-making) could also help reduce stigma around suicidality and lower burnout risks among HCPs.

Unlike prior studies (Valente and Saunders 2004; Wardig et al. 2022), we found effective referral systems and strong collaboration with the departments (as it is a standard in German certified cancer centers). Networking with psycho-oncologists and psychiatric clinics emerged as a valuable resource to manage suicidal statements effectively, which echoes findings by Granek et al. (2019b).

### Limitations

Although diverse in terms of age, gender, and professional group, this study contains a small sample. Due to the sample size, the results must be interpreted with caution. They provide valuable insights, but cannot be generalised across contexts and will be validated by a currently running large survey. The results might thus be particularly representative of working in a large, certified cancer center. Not all professional groups involved in caring for individuals with cancer, such as social workers and nutritional counselors, could be represented as we could not recruit them to participate in the study (whether due to lack of time, motivation, or looser professional relationships). Additionally, the sample was not gender-balanced, reflecting the overrepresentation of women among HCPs. Participant self-selection and potential social desirability biases must also be considered, as those more interested in the topic may have been more likely to participate. As such, based on this study, the challenges HCPs face might still be underestimated. Moreover, we could not assess actual behavior, but relied on self-reports which could be influenced by recall bias. Furthermore, some questionnaires were not completed immediately after the interviews due to the limited time available to the participants, which may also have led to biases. Future studies are needed to validate and enrich the present information (involving HCPs from other regions and settings). Building on the present work, a large, international survey with a quantitative focus is currently ongoing (to validate the findings across healthcare contexts and settings and to expand on them); also because the present quantitative analyses are limited by the small sample size implicating low power to detect relevant associations.

## Conclusion

This mixed-methods study represents the first of its kind in Germany, combining a theory- and evidence-driven approach with insights from HCPs’ daily experiences. It reveals that while HCPs encounter diverse manifestations of suicidality, they often struggle with the aim of an active suicide exploration and do not follow a consistent approach as their task. The study also highlights differences across professional groups, age, and gender. The various aspects that have previously been found to shape responses to suicidal patients could be improved by the implementation of guidelines, suicide prevention training, supervision, and specific screening. Thus, the findings can inform those practically applicable solutions to strengthen HCPs and enhance care and suicide prevention for cancer patients.

## Electronic supplementary material

Below is the link to the electronic supplementary material.

Supplementary Material 1

## Data Availability

The full interview transcripts cannot be made available as they contain sensitive information about healthcare professionals and their patients. The study aims and procedure were preregistered as part of a larger, mixed-methods project via the Open Science Framework where the materials are made available as well (https://osf.io/4c6jr).

## References

[CR1] Amiri S, Behnezhad S (2019) Cancer diagnosis and suicide mortality: a systematic review and Meta-analysis. Arch Suicide Res 1–19. 10.1080/13811118.2019.1596182 10.1080/13811118.2019.1596182 30955459

[CR2] Appleby L, Kapur N, Shaw J, Hunt IM, Gianatsi M, Turnbull P, Rodway C, Tham SG, Burns J, Richards N (2018) National confidential inquiry into suicide and homicide–annual report: England, Northern Ireland, Scotland and Wales. *University of Manchester*

[CR3] Beierlein C, Kemper C, Kovaleva A, Rammstedt B (2013) Kurzskala Zur Erfassung Allgemeiner Selbstwirksamkeitserwartungen (ASKU). Methoden Daten Analysen (mda) 7(2):251–278

[CR4] Bordin ES (1979) The generalizability of the psychoanalytic concept of the working alliance. Psychotherapy: Theory Res Pract 16(3):252–260

[CR5] Calear AL, Batterham PJ (2019) Suicidal ideation disclosure: patterns, correlates and outcome. Psychiatry Res 278:1–6. 10.1016/j.psychres.2019.05.024 31128420 10.1016/j.psychres.2019.05.024

[CR6] Corbin J, Strauss A (1990) Grounded Theory Research - procedures, canons and evaluative criteria. Z Fur Soziologie 19(6):418–427

[CR7] D’Alton P, O’Meara R, Langford S, McDonnell Z, Nuzum A, Murthy VE, Craddock F, Cogley C, McCormack D (2021) Barriers to cancer care for people with significant mental health difficulties: what healthcare staff say? Psychooncology 30(12):2032–2038. 10.1002/pon.5790 34453853 10.1002/pon.5790

[CR8] Demesmaeker A, Baelde N, Amad A, Roche J, Playe M, Vaiva G, Amariei A, Blervaque W, Defebvre MM, Caron B, Puisieux F, Plancke L (2023) Assessment of a Suicide Prevention Gatekeeper Training Program for Nursing Home Staff. J Geriatr Psychiatr Neurol 36(4):309–315. 10.1177/08919887221149142 10.1177/08919887221149142 36594410

[CR9] Destatis SB (2024) *Todesursachen Suizide*. https://www.destatis.de/DE/Themen/Gesellschaft-Umwelt/Gesundheit/Todesursachen/Tabellen/suizide.html

[CR10] Deutsche Krebsgesellschaft; Deutsche Krebshilfe; AWMF (2023) Psychoonkologische Diagnostik, Beratung und Behandlung von erwachsenen Krebspatient*innen Version 2.1 – August 2023 AWMF-Registernummer: 032-051OL

[CR11] Dillon CB, Saab MM, Meehan E, Goodwin MJ, Murphy M, Heffernan MS, Greaney MS, Kilty C, Hartigan I, Chambers D, Twomey U, Horgan A (2020) Staff awareness of suicide and self-harm risk in healthcare settings: a mixed-methods systematic review. J Affect Disord 276:898–906. 10.1016/j.jad.2020.07.113 32739708 10.1016/j.jad.2020.07.113

[CR12] Du L, Shi HY, Qian Y, Jin XH, Li Y, Yu HR, Liu XM, Fu XL, Chen HL (2020) Association between social support and suicidal ideation in patients with cancer: a systematic review and meta-analysis. Eur J Cancer Care (Engl) e13382. 10.1111/ecc.13382 10.1111/ecc.13382 33277772

[CR13] Ernst M, Schwinn T, Hirschmiller J, Cleare S, Robb KA, Brahler E, Zwerenz R, Wiltink J, O’Connor RC, Beutel ME (2024) To what extent are psychological variables considered in the study of risk and protective factors for suicidal thoughts and behaviours in individuals with cancer? A systematic review of 70 years of research. Clin Psychol Rev 109:102413. 10.1016/j.cpr.2024.102413 38518584 10.1016/j.cpr.2024.102413

[CR14] Ferracioli NGM, Rodrigues ECG, Santos MAD (2023) Bittersweet transformative experiences in professionals working with suicidal patients: a meta-synthesis. Braz J Psychiatry 45(1):62–70. 10.47626/1516-4446-2022-2617 35881567 10.47626/1516-4446-2022-2617 PMC9976915

[CR15] Fluckiger C, Del Re AC, Wampold BE, Horvath AO (2018) The alliance in adult psychotherapy: a meta-analytic synthesis. Psychother (Chic) 55(4):316–340. 10.1037/pst0000172 10.1037/pst0000172 29792475

[CR16] Frey LM, Hans JD, Cerel J (2016) Perceptions of suicide stigma. Crisis 37(2):95–103. 10.1027/0227-5910/a000358 26695868 10.1027/0227-5910/a000358

[CR17] Granek L, Nakash O, Ariad S, Shapira S, Ben-David M (2018) Oncologists’ identification of mental health distress in cancer patients: Strategies and barriers. Eur J Cancer Care (Engl) 27(3). 10.1111/ecc.12835 10.1111/ecc.12835 29508452

[CR18] Granek L, Nakash O, Ariad S, Shapira S, Ben-David M (2019a) Cancer patients’ Mental Health Distress and Suicidality. Crisis 40(6):429–436. 10.1027/0227-5910/a000591 31030550 10.1027/0227-5910/a000591

[CR19] Granek L, Nakash O, Ariad S, Shapira S, Ben-David M (2019b) Strategies and barriers in addressing Mental Health and Suicidality in patients with Cancer. Oncol Nurs Forum 46(5):561–571. 10.1188/19.ONF.561-571 31424452 10.1188/19.ONF.561-571

[CR20] Groth T, Boccio DE (2019) Psychologists’ willingness to provide services to individuals at risk of suicide. Suicide Life Threat Behav 49(5):1241–1254. 10.1111/sltb.12501 30091151 10.1111/sltb.12501

[CR21] Guest G, Bunce A, Johnson L (2006) How many interviews are Enough? Field Methods 18(1):59–82. 10.1177/1525822x05279903

[CR22] Hawton K, Knipe D, Pirkis J (2024) Restriction of access to means used for suicide. Lancet Public Health 9(10):e796–e801. 10.1016/S2468-2667(24)00157-9 39265608 10.1016/S2468-2667(24)00157-9

[CR23] Hom MA, Stanley IH, Podlogar MC, Joiner TE Jr. (2017) Are you having thoughts of suicide? Examining experiences with disclosing and denying suicidal ideation. J Clin Psychol 73(10):1382–1392. 10.1002/jclp.22440 28085200 10.1002/jclp.22440

[CR24] Hooper LM, Epstein SA, Weinfurt KPD, Qu J, L., Hannah NJ (2012) Predictors of Primary Care Physicians’ Self-reported intention to Conduct suicide risk assessments. J Behav Helath Serv Res 39(2):103–115. 10.1007/s11414-011-9268-5 10.1007/s11414-011-9268-5 PMC3586785 22218814

[CR25] Jerant A, Duberstein P, Cipri C, Bullard B, Stone D, Paterniti D (2019) Stakeholder views regarding a planned primary care office-based interactive multimedia suicide prevention tool. Patient Educ Couns 102(2):332–339. 10.1016/j.pec.2018.09.007 30220599 10.1016/j.pec.2018.09.007 PMC6886248

[CR26] Jobes DA, Barnett JE (2024) Evidence-based care for suicidality as an ethical and professional imperative: how to decrease suicidal suffering and save lives. Am Psychol. 10.1037/amp0001325 38695782 10.1037/amp0001325

[CR27] Kuckartz U (2010) Einführung in die computergestützte Analyse Qualitativer Daten, vol 3. Springer

[CR28] Kuckartz U, Rädiker S (2022) *Qualitative Inhaltsanalyse. Methoden, Praxis, Computerunterstützung* (Vol. 5. Auflage). Beltz Juventa

[CR29] Lauterbach E, Kienast T (2009) Suizidalität. *Psychiatrie und Psychotherapie up2date*, *3*(03), 197–212

[CR30] Lengvenyte A, Olie E, Strumila R, Navickas A, Gonzalez Pinto A, Courtet P (2021) Immediate and short-term efficacy of suicide-targeted interventions in suicidal individuals: a systematic review. World J Biol Psychiatry 22(9):670–685. 10.1080/15622975.2021.1907712 33783294 10.1080/15622975.2021.1907712

[CR31] Ludwig J, Liebherz S, Dreier M, Harter M, von dem Knesebeck O (2020) [The stigma of suicide scale: psychometric validation of the German short version (SOSS-SF-D)]. Psychiatr Prax 47(8):433–439. 10.1055/a-1145-3992(Die Stigma of Suicide Scale: psychometrische Uberprufung der deutschen Kurzversion (SOSS-SF-D).) 32588402 10.1055/a-1145-3992

[CR32] Luoma JB, Martin CE, Pearson JL (2002) Contact with mental health and primary care providers before suicide: a review of the evidence. Am J Psychiatry 159(6):909–916. 10.1176/appi.ajp.159.6.909 12042175 10.1176/appi.ajp.159.6.909 PMC5072576

[CR33] McCabe R, Sterno I, Priebe S, Barnes R, Byng R (2017) How do healthcare professionals interview patients to assess suicide risk? BMC Psychiatry 17(1):122. 10.1186/s12888-017-1212-7 28372553 10.1186/s12888-017-1212-7 PMC5379679

[CR34] Miller M, Mogun H, Azrael D, Hempstead K, Solomon DH (2008) Cancer and the risk of suicide in older americans. J Clin Oncol 26(29):4720–4724. 10.1200/JCO.2007.14.3990 18695256 10.1200/JCO.2007.14.3990

[CR35] Muehlenkamp JJ, Grande N, Talbott M (2023) Evidence-based vs Informal suicide training: nurse confidence and comfort with suicidal patient care. J Emerg Nurs 49(2):266–274. 10.1016/j.jen.2022.12.003 36599734 10.1016/j.jen.2022.12.003

[CR36] Nationaler Krebsplan (2017) Nationaler Krebsplan Handlungsfelder, Ziele Und Umsetzungsempfehlungen. Bundesministerium für Gesundheit

[CR37] Nuij C, van Ballegooijen W, de Beurs D, Juniar D, Erlangsen A, Portzky G, O’Connor RC, Smit JH, Kerkhof A, Riper H (2021) Safety planning-type interventions for suicide prevention: meta-analysis. Br J Psychiatry 219(2):419–426. 10.1192/bjp.2021.50 35048835 10.1192/bjp.2021.50

[CR38] O’Conner RC (2016) *The International Handbook of Suicide Prevention*. 10.1002/9781118903223

[CR39] O’Connor RC (2021) When it is darkest: why people die by suicide and what we can do to prevent it. Random House

[CR40] Osteen P, Frey JM, Woods MN, Ko J, Shipe S (2017) Modeling the Longitudinal Direct and Indirect effects of attitudes, Self-Efficacy, and behavioral intentions on practice behavior outcomes of suicide intervention training. Suicide Life Threat Behav 47(4):410–420. 10.1111/sltb.12288 27539239 10.1111/sltb.12288

[CR41] Ozturk S, Hicdurmaz D (2023) A qualitative study on the perspectives and needs of oncology nurses about recognition and management of suicide risk in cancer patients. J Clin Nurs 32(5–6):749–763. 10.1111/jocn.16304 35343003 10.1111/jocn.16304

[CR42] Pendergrass JC, Targum SD, Harrison JE (2017) Cognitive impairment Associated with Cancer: a brief review. Innovations Clin Neurosci 15(1–2):36–44 PMC5819720 29497579

[CR43] Posner K, Brown GK, Stanley B, Brent DA, Yershova KV, Oquendo MA, Currier GW, Melvin GA, Greenhill L, Shen S, Mann JJ (2011) The Columbia–suicide severity rating scale: initial validity and internal consistency findings from three Multisite studies with adolescents and adults. Am J Psychiatry 168(12):1266–1277. 10.1176/appi.ajp.2011.10111704 22193671 10.1176/appi.ajp.2011.10111704 PMC3893686

[CR44] Riblet NB, Matsunaga S, Lee Y, Young-Xu Y, Shiner B, Schnurr PP, Levis M, Watts BV (2022) Tools to detect risk of death by suicide: a systematic review and Meta-analysis. J Clin Psychiatry 84(1). 10.4088/JCP.21r14385 10.4088/JCP.21r14385 PMC9890591 36383739

[CR45] Robson A, Scrutton F, Wilkinson L, MacLeod F (2010) The risk of suicide in cancer patients: a review of the literature. Psychooncology 19(12):1250–1258. 10.1002/pon.1717 20213857 10.1002/pon.1717

[CR46] Saini P, Hunt A, Blaney P, Murray A (2024) Recognising and responding to suicide-risk factors in primary care: a scoping review. J Prev 2022. 10.1007/s10935-024-00783-1 10.1007/s10935-024-00783-1 PMC11493792 38801507

[CR47] Schellekens MPJ, Wolvers MDJ, Schroevers MJ, Bootsma TI, Cramer AOJ, van der Lee ML (2020) Exploring the interconnectedness of fatigue, depression, anxiety and potential risk and protective factors in cancer patients: a network approach. J Behav Med 43(4):553–563. 10.1007/s10865-019-00084-7 31435892 10.1007/s10865-019-00084-7 PMC7366596

[CR48] Schwinn T, Paul RH, Hirschmiller J, Brahler E, Wiltink J, Zwerenz R, O’Connor RC, Wild PS, Munzel T, Konig J, Geschke K, Moehler M, Konstantinides S, Justenhoven C, Lackner KJ, Pfeiffer N, Beutel ME, Ernst M (2024) Prevalence of current suicidal thoughts and lifetime suicide attempts in individuals with cancer and other chronic diseases in Germany: evidence for differential associations from a representative community cohort. J Affect Disord 367:193–201. 10.1016/j.jad.2024.08.093 39178957 10.1016/j.jad.2024.08.093

[CR49] Senf B, Maiwurm P, Fettel J (2020) Exposure to suicidality in professionals working with oncology patients: an online survey. Psychooncology 29(10):1620–1629. 10.1002/pon.5479 32672869 10.1002/pon.5479

[CR50] Senf B, Maiwurm P, Fettel J (2022) Attitudes and opinions towards suicidality in professionals working with oncology patients: results from an online survey. Support Care Cancer 30(2):1775–1786. 10.1007/s00520-021-06590-2 34599381 10.1007/s00520-021-06590-2 PMC8727409

[CR51] Siau CS, Wee L-H, Ibrahim N, Visvalingam U, Yeap LLL, Wahab S (2018) Gatekeeper suicide training’s effectiveness among Malaysian Hospital Health professionals: a Control Group Study with a three-Month Follow-Up. J Continuing Educ Health Professions 38(4):227–234. 10.1097/ceh.0000000000000213 10.1097/CEH.0000000000000213 30036213

[CR52] Solin P, Tamminen N, Partonen T (2021) Suicide prevention training: self-perceived competence among primary healthcare professionals. Scand J Prim Health Care 39(3):332–338. 10.1080/02813432.2021.1958462 34340646 10.1080/02813432.2021.1958462 PMC8475147

[CR53] Stuber J, Massey A, Payn B, Porter S, Ratzliff A (2023) Training Health Care professionals in suicide Assessment, Management, and treatment. Psychiatr Serv 74(1):88–91. 10.1176/appi.ps.202100571 35734862 10.1176/appi.ps.202100571

[CR54] Trevino KM, Abbott CH, Fisch MJ, Friedlander RJ, Duberstein PR, Prigerson HG (2014) Patient-oncologist alliance as protection against suicidal ideation in young adults with advanced cancer. Cancer 120(15):2272–2281. 10.1002/cncr.28740 24888503 10.1002/cncr.28740 PMC4356118

[CR55] Turecki G, Brent DA, Gunnell D, O’Connor RC, Oquendo MA, Pirkis J, Stanley BH (2019) Suicide and suicide risk. Nat Reviews Disease Primers 5(1). 10.1038/s41572-019-0121-0 10.1038/s41572-019-0121-0 31649257

[CR56] Turner K, Stover AM, Tometich DB, Geiss C, Mason A, Nguyen OT, Hume E, McCormick R, Powell S, Hallanger-Johnson J, Patel KB, Kirtane KS, Jammigumpula N, Moore C, Perkins R, Rollison DE, Jim HSL, Oswald LB, Crowder S, Gonzalez BD, Robinson E, Tabriz AA, Islam JY, Gilbert SM (2023) Oncology Providers’ and professionals’ experiences with suicide risk screening among patients with Head and Neck Cancer: a qualitative study. JCO Oncol Pract 19(6):e892–e903. 10.1200/OP.22.00433 36395441 10.1200/OP.22.00433 PMC10337750

[CR57] Valente S, Saunders JM (2004) Barriers to suicide risk management in clinical practice: a national survey of oncology nurses. Issues Ment Health Nurs 25(6):629–648. 10.1080/01612840490472147 15371147 10.1080/01612840490472147

[CR58] Wardig RE, Hultsjo S, Lind M, Klaveback I (2022) Nurses’ experiences of Suicide Prevention in Primary Health Care (PHC) - a qualitative interview study. Issues Ment Health Nurs 43(10):903–912. 10.1080/01612840.2022.2089789 35793075 10.1080/01612840.2022.2089789

[CR59] Wee LH, Ibrahim N, Wahab S, Visvalingam U, Yeoh SH, Siau CS (2020) Health-Care workers’ perception of patients’ suicide intention and factors leading to it: a qualitative study. Omega (Westport) 82(2):323–345. 10.1177/0030222818814331 30482086 10.1177/0030222818814331

[CR60] World Health Organization (2024) *Suicide*. Retrieved from https://www.who.int/news-room/fact-sheets/detail/suicide

[CR61] Zaorsky NG, Zhang Y, Tuanquin L, Bluethmann SM, Park HS, Chinchilli VM (2019) Suicide among cancer patients. Nat Commun 10(1):207. 10.1038/s41467-018-08170-1 30643135 10.1038/s41467-018-08170-1 PMC6331593

