# Supplementary material for: Practitioners’ perspective: a mixed-methods study on dealing with suicidality from the perspective of oncological healthcare professionals
Source: J Cancer Res Clin Oncol. 2025 Jan 28;151(2):54. doi: 10.1007/s00432-025-06106-z (PMC11775075; doi:10.1007/s00432-025-06106-z)
Supplement: Supplementary file 1 — Supplementary Material 1 [file 432_2025_6106_MOESM1_ESM.docx]

**Supplements**

Supplementary Table 1. Category system

| Category | Theme | Subtheme |
| --- | --- | --- |
| Experiences | Negative | Gradient from passive death wishes to suicidal thoughts |
|  |  | Stopping life-sustaining actions/suicidal actions and suicide deaths |
|  |  | Conversations about death and dying |
|  | Positive | Positive experiences |
| Exploration of suicidality | No active exploration |  |
|  | Exploration in line with one's gut feeling | Explicit questions |
|  |  | Non-explicit questions |
| Management | After a suicide | Reaction: Unpleasant feelings vs. Blunting |
|  |  | Debriefing in the team vs. no debriefing |
|  |  | Discussion with focus on future prevention vs. wrong procedure in the past |
|  | After expressions of suicidality | Contact persons and forwarding |
|  |  | Strategies |

Supplementary Table 2. Professionals' reported reasons for the exploration of suicidality in their patients (drawn from interview and questionnaire data).

|  | When do professionals explore suicidality? | | | | | |
| --- | --- | --- | --- | --- | --- | --- |
| Source of information | (a) when the patient expresses death wishes | (b) When the patient scores high on distress/depression (in screening instruments) | (c) Unspecific information  (e.g., one’s gut feeling) | (d) More specific criteria (e.g., patients' mood, noting a lack of social support) | Professionals report exploring suicidality routinely without further explanation | Professionals report *not* exploring suicidality without further explanation |
| Interview | 9 | 3 | 4 | 1 | 1 | 5 |
| Questionnaire | 6 | 4 | 6 | 3 | 0 | 7 |
